# Phage-Microbiota Crosstalk: Implications for Central Nervous System Disorders

**DOI:** 10.3390/ijms26136183

**Published:** 2025-06-26

**Authors:** Valentina Salari, Edoardo Parrella, Francesca Mengoni, Laís Cintra, Giuseppe Bertini, Paolo Francesco Fabene

**Affiliations:** 1 Section of Innovation Biomedicine, Department of Engineering for Innovation Medicine, University of Verona, 37134 Verona, Italy; valentina.salari@univr.it (V.S.); edoardo.parrella@unicamillus.org (E.P.); francesca.mengoni@univr.it (F.M.); 2 Departmental Faculty of Medicine, Saint Camillus International University of Health Sciences, 00131 Rome, Italy; 3 Section of Anatomy and Histology, Department of Neurosciences, Biomedicine and Movement Sciences, Faculty of Medicine, University of Verona, 37134 Verona, Italy; laisncintra@gmail.com (L.C.); giuseppe.bertini@univr.it (G.B.)

**Keywords:** phages, bacteriophages, bacteria, microbiota, gut, central nervous system (CNS), brain

## Abstract

The gut microbiota constitutes a complex community of microorganisms (including bacteria, viruses, fungi, and protozoa) within the intestinal tract. Over the years, an increasing number of studies have highlighted the bidirectional communication between the gut microbiota and the central nervous system (CNS), a relationship commonly referred to as the “microbiota–gut–brain axis”. In particular, the crosstalk between the gut microbiota and the brain has been associated with the pathogenesis and progression of various CNS disorders. Phages, or bacteriophages, viruses that specifically infect bacteria, constitute the most abundant viral component within the gut microbiota. However, despite their abundance and significance in the gut microbial community, studies exploring the relationship between phages and the CNS remain surprisingly limited. This review examines the biological interplay between gut-resident phages and the CNS. Furthermore, we discuss the current literature linking phages to CNS-related pathologies.

## 1. Introduction

The term “gut microbiota” refers to the community of microorganisms (bacteria, archaea, viruses, and eukaryotes) that inhabit the gastrointestinal tract. This complex ecosystem influences the host during homeostasis and diseases [1]. Notably, over the past decade, accumulating evidence has proven the presence of bidirectional communication between the gut microbiome and the central nervous system (CNS), termed the “microbiota–gut–brain–axis” [2,3]. Different direct and indirect pathways have been proposed to describe this communication, including modulation of the immune system, vagus nerve, enteric nervous system, neuroendocrine system, neurotransmitters, and release of metabolites or neuroactive substances in the circulatory system [2].

Nevertheless, even though gut microbiota includes bacteria, archaea, viruses, and eukaryotes, most studies have focused mainly on bacterial populations in the past decade. The emphasis on bacteria results from their importance in the gut microbiota regarding microbial DNA content, cell numbers, and overall microbial biomass [4,5,6]. Also, since a few years ago, the method employed to characterize the microbiome was the 16S rRNA gene sequencing (16Ss), which targeted a single gene found exclusively in bacteria and archaea. However, recent advances in high-throughput sequencing technologies enabled the investigation of the profile of bacteria, fungi, viruses, and many other types of microorganisms simultaneously [7].

In addition to bacteria, a high amount of viruses inhabit the human gut, and they are collectively referred to as the virome [4]. Among these, bacteriophages (phages), viruses that exclusively infect bacteria, represent the most abundant viral components within the human body [8,9] and are thought to play an important role in the structure and function of microbial communities [10]. Recently, bacteriophages have gained attention as an effective strategy for microbiota modulation due to their high specificity, self-limiting characteristics, and potential to overcome antibiotic resistance [11]. Unlike broad-spectrum antibiotics, which target bacteria indiscriminately, phages selectively act on specific bacterial strains, preserving microbiome diversity and functionality [12,13,14].

**Figure 1:**
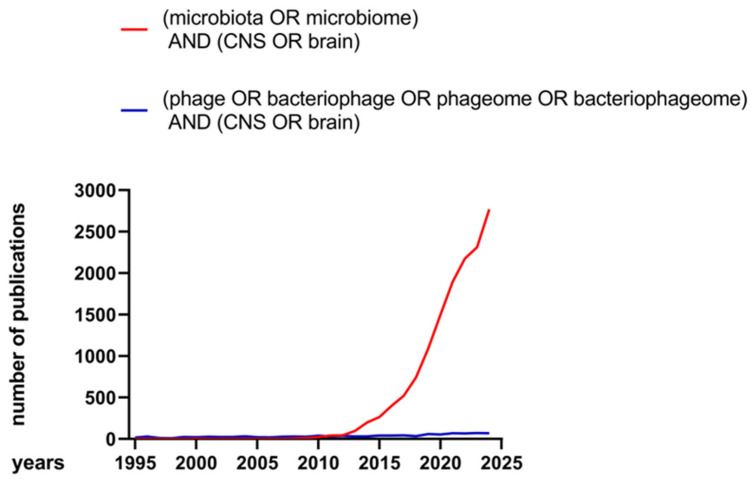
Number of scientific publications investigating microbiota and CNS, or phages and CNS, between 1995 and 2024.

Despite the abundance of viruses, and phages in particular, in the microbiota composition, surprisingly few studies investigate the impact of phages on the CNS. Using the PubMed database, a phrase search of (“microbiota” OR “microbiome”) AND (“CNS” OR “brain”) in the past 30 years (range 1995–2024) showed a dramatic increase in publications over the past decade (Figure 1). Conversely, the phrase search (“phage” OR “bacteriophage” OR “phageome” OR “bacteriophageome”) AND (“CNS” OR “brain”) yielded a number of publications far lower. For example, in 2024, 2769 studies were published on the microbiota, but only 68 on phages (Figure 1).

In this review, we aim to highlight the potential role of phages as specific microbiome modulators, providing a promising future therapeutic strategy for CNS disorders.

## 2. Phages and Their Presence in the Human Body

Phages are primarily classified based on their morphology and genomic sequences [15,16,17]. Furthermore, depending on their life cycle, phages are categorized as either virulent (also known as strictly lytic phages) or temperate (also called lysogenic phages) [18,19,20,21]. The infection process begins when a phage recognizes and binds to receptors on the surface of the host bacteria. Once the phage injects its DNA into the bacteria, virulent and temperate phages follow different paths. Virulent phages can only follow a lytic cycle, where they use the host cell machinery to produce new phage particles and eventually cause the lysis of the host cell, releasing the new infectious phage particles.

On the other hand, temperate phages can follow a lytic or lysogenic cycle after infection, depending on factors like bacterial density and environmental stress. In the lysogenic cycle, temperate phages integrate their genomes into the host bacterial chromosomes, a process known as lysogenization, and exist as prophages, replicating along with the host′s genetic material. Temperate phages can switch to the lytic cycle if their genome is excised from the host genome, either spontaneously or in response to environmental stress, leading to the host cell’s lysis and the release of phage particles.

Phages are found in all human body niches [22], including the gut [23,24,25], skin [26,27], oral cavity [28,29,30], lungs [31,32], and urogenital tract [33,34]. Most of these phages are temperate, meaning they can either integrate their DNA into bacterial genomes as prophages or persist extrachromosomally as episomes, genetic elements that replicate independently of the host chromosome. In both forms, they can alter the phenotype of the host bacteria through lysogenic conversion [23,35]. Metagenomic analyses have shown the presence of phages even in the blood [36,37,38]. Circulating in the bloodstream, these phages can interact with immune cells and trigger innate and adaptive immune responses [39].

Among the bacterial communities present in the human body, the one in the gut is by far the largest and most complex [40,41]. Likewise, the most represented community of phages in the human body is also found in the gut, where they form a significant part of the intestinal microbiota [24,42,43,44]. Most phages identified in the human gut belong to the order Caudovirales, which includes five families: Ackermannviridae, Herelleviridae, Myoviridae, Podoviridae and Siphoviridae [45].

Interestingly, changes in the composition of the intestinal bacterial population also lead to changes in the phage community, suggesting that the diversity of phages and the diversity of bacteria in the gut are closely linked [46,47]. In light of the abundance and the not fully understood role of intestinal phages, in this review, we have focused on the phages residing in the gut.

## 3. Therapeutic Use of Phages

Phage therapy has gained emphasis in the last decade due to its high specificity, particularly against pathogenic/multidrug-resistant bacteria [48,49]. Indeed, in contrast to fecal microbiota transplantation, which modulates the recipient patients′ entire microbial community [50,51], phage therapy can target specific bacterial strains, preserving microbiome diversity and functionality. Although researchers have demonstrated the safety of phage therapy [52], many barriers to its wide application remain. First, the high specificity of bacteriophages for individual bacterial strains poses a challenge for their use in polymicrobial infections or when the exact pathogen has not been identified. Second, most current studies on phage therapy are predominantly conducted in animal models, necessitating further validation in human clinical settings. Lastly, a key concern is the possibility that lytic phages would turn to a temperate state after administration in humans, thus reducing their effectiveness.

The implementation of phage therapy involves multiple steps: (i) phage characterization; (ii) susceptibility test; (iii) phage propagation; (iv) phage purification and quality control; (v) clinical applications; and (vi) therapeutic monitoring [53]. Various delivery strategies have been developed to effectively target bacterial cells, including conventional phage therapy, phage-derived enzymes, immuno-phage-antibiotic synergy, and bioengineered phages [54]. 

Delivery methods are adapted to the patient and infection site. Systemic infections are typically treated with intravenous (i.v.) administration, while local infections benefit from site-specific methods, like nebulization for respiratory infections, intravesicular delivery for urinary tract infections, intra-articular injections for joint infections, and topical applications for skin wounds. Local delivery can achieve higher phage concentrations at the target site compared to i.v. delivery. Some studies suggest that combining systemic and local approaches may enhance therapeutic outcomes [53].

Despite its promise, phage therapy faces several challenges, including meeting current quality and safety standards, ensuring the long-term stability of phage preparations, developing effective phage screening assays, and overcoming the limited efficacy of phages in biofilms [55]. Additional hurdles include preventing or managing bacterial resistance, avoiding neutralization by host antibodies, and addressing phage clearance by the liver and spleen. Establishing a consistent regulatory framework for phage-based products also remains a critical need [56].

Clinical data show that bacterial resistance poses a major limitation to phage therapy (50% of sepsis cases involve phage-resistant bacteria) [54,57]. Resistance mechanisms include spontaneous resistance caused by the lack of functional surface receptors required for phage adsorption, biofilm-mediated barriers through the production of exopolysaccharides, and acquired resistance *via* horizontal gene transfer (through plasmids or temperate phages, which can carry genes for antibiotic and phage resistance) [54].

To address these issues, phage therapy commonly employs cocktails—combinations of multiple phages—to reduce the chance that bacteria will simultaneously develop resistance to all components. This multi-targeted approach increases the robustness and potential success of treatment [54,55]. On the other hand, designing effective phage cocktails brings its own complications, such as insufficient receptor coverage, cross-resistance among phages, and inadequate phage concentrations that allow resistant subpopulations to emerge [53]. Promising recent approaches involve combining phages that target multiple, non-redundant bacterial receptors. However, resistance can still develop, necessitating complementary strategies—such as exploiting evolutionary trade-offs like reduced virulence or restored antibiotic sensitivity in phage-resistant bacteria [53]. 

## 4. The Microbiota–Gut–Brain Axis

The microbiota–gut–brain axis (MGBA) is a complex, bidirectional communication network linking the gut microbiota with the CNS through neural, endocrine, immune, and metabolic pathways [58,59,60,61,62,63,64,65,66,67].

The vagus nerve represents the most direct neuroanatomical pathway connecting the gut and the enteric nervous system (ENS) to the brain and the hypothalamic–pituitary–adrenal (HPA) axis [68,69]. Although vagal afferent fibers terminate in various layers of the intestinal wall, they cannot directly access the gut microbiota, as they do not penetrate the intestinal epithelium. Different mechanisms have been proposed by which the vagus nerve detects signals from the gut microbiota, including direct microbial recognition and indirect pathways mediated by enteroendocrine cells [69]. Indeed, vagal afferent fibers can be directly activated by microbiota metabolites such as short-chain fatty acids (SCFAs) [70] or by lipopolysaccharide (LPS) through toll-like receptor 4 (TLR4) [71]. Besides the direct pathway, the vagal nerve may receive intestinal microbiota information through enteroendocrine cells. These cells can detect bacterial products in the intestinal lumen and secrete several peptides that can activate vagal afferent neurons [72,73,74]. Similarly, enterochromaffin cells, a subtype of enteroendocrine cells, can be stimulated by microbial metabolites to release serotonin, activating the vagus nerve [75].

Gut microorganisms produce a variety of bioactive molecules that can influence the CNS. As discussed above, some of these molecules act at the intestinal level on the vagus nerve and the enteroendocrine cells. In contrast, others can cross the intestinal barrier, enter the systemic circulation, and potentially reach the CNS by crossing the blood-brain barrier (BBB) [2].

SCFAs are the primary metabolites produced by gut microbiota in the large intestine through the anaerobic fermentation of indigestible polysaccharides. SCFAs may impact gut–brain communication and brain function either directly or indirectly [76,77]. At the intestinal level, SCFAs can directly or indirectly activate the vagus nerve as previously described, while modulating systemic inflammation by promoting the differentiation of T regulatory cells (Tregs) and regulating interleukin secretion [78]. However, colon-derived SCFAs can also enter the systemic circulation, cross the BBB, and reach the brain, where they can modulate neuroinflammation and neuronal homeostasis and function [76,77].

Some metabolites produced by gut bacteria play crucial roles in neurotransmitter metabolism. For example, *Clostridium sporogenes* contribute to the conversion of tryptophan into tryptamine [79], a neuromodulator and neurotransmitter that stimulates serotonin and dopamine release [79]. *Lactobacillus* and *Bifidobacterium* species facilitate the conversion of glutamate into gamma-aminobutyric acid [80], a key neurotransmitter.

Furthermore, gut dysbiosis can trigger the release of pro-inflammatory derivatives (LPS and peptidoglycan) that promote the release of cytokines (IL-1β, IL-6, and TNF-α) [81,82,83]. In addition to its effect on the vagus nerve, LPS and the released cytokines can cross the BBB and activate the HPA axis [84].

Finally, it is important to note that numerous molecules produced by the microbiota can influence the integrity of both the gut barrier and the BBB. For example, changes in the gut microbiota population have been associated with increased cortisol production, which in turn raises intestinal permeability by disrupting tight junctions [61,81]. Studies in mice have demonstrated that some microorganisms play a crucial role in maintaining BBB integrity by regulating junction proteins such as occludin and claudin-5 [50,69]. In germ-free mice, the production of these proteins was reduced by up to 75% [85], highlighting the microbiota′s impact on barrier function. The disruption of the gut barrier and the BBB can facilitate the translocation of microbial products and inflammatory mediators into systemic circulation and the brain, potentially contributing to chronic inflammation, neuroinflammation, and, ultimately, contributing to neuronal cell damage [81,86,87].

## 5. Phages and the CNS

**Figure 2:**
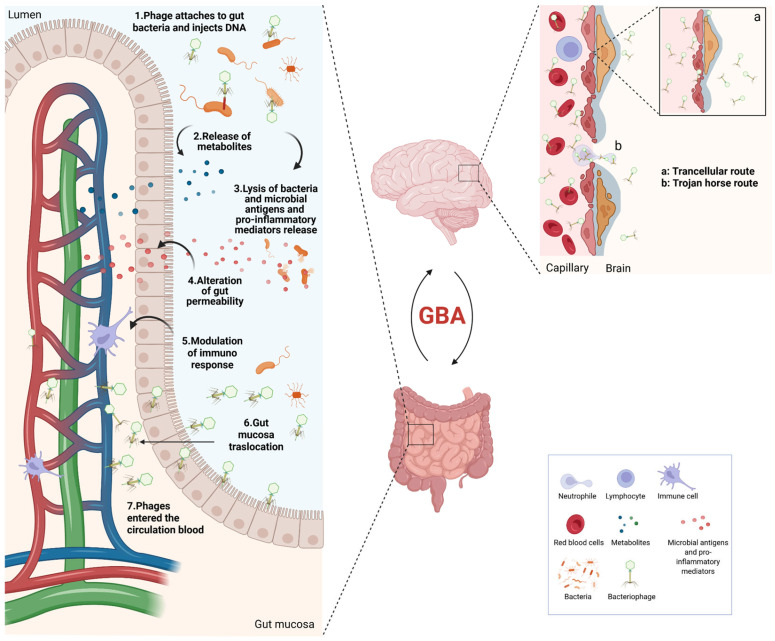
Possible interactions between gut phages and CNS. (1) Phages can directly modulate bacteria through (2) the lysogenic cycle, leading to the release of several bacteria metabolites, or (3) the lytic cycle, causing bacteria lysis and the release of microbial antigens and pro-inflammatory mediators. This, in turn, can lead to (4) altered gut permeability and (5) activation of the immune response, facilitating (6) phage translocation through the gut mucosa and (7) their entry into the bloodstream by crossing the epithelial cell barrier. Phages may cross the neural endothelial cells via (a) the transcellular route or (b) the Trojan horse mechanism. Created with BioreRender.com.

It has been hypothesized that gut phages may influence CNS health in different ways [43,88,89,90], as illustrated in Figure 2.

First, gut phages can directly shape the gut bacterial community structure [11,43,91,92,93,94]. As natural predators of bacteria, bacteriophages influence the gut bacterial community through a predator–prey dynamic, thus contributing to the regulation of bacterial metabolism and vitality [95,96]. For example, phage-dependent lysis of bacterial species within a microbial community fosters microbial diversity and evolution while optimizing the community′s resource usage efficiency [97,98]. On the other hand, lysogeny can affect the bacterial community composition by providing indirect benefits to the lysogens (i.e., prophage-carrying bacteria) and through horizontal gene transfer of beneficial genes between host bacteria [91,99,100]. An imbalance between lysis and lysogeny at the intestinal level and an altered spatial distribution of phages appears to be linked to pathological conditions such as inflammatory bowel disease and leukemia [101,102,103].

Besides their direct effects on bacterial populations, phages can indirectly affect bacterial colonization through the gut mucosal surface [25,88]. The gut mucosal surface is mainly composed of mucin glycoproteins secreted by intestinal epithelium and represents a critical environment involved in the interaction of microbiota–host organisms [104]. Indeed, mucus layers provide structural support and nutrients for gut microbiota and shape the microbiota by selecting commensal symbionts [105]. Different families of phages in the mucosal surface layer can bind to glycan residues present on mucin glycoproteins by their capsid proteins, such as immunoglobulin (Ig)–like domains [106]. The interaction between phages and the mucosal surface may promote opposing effects. Phage adherence to the mucus layer can form an antimicrobial barrier that inhibits bacterial attachment and colonization, limiting the diffusion of bacteria through the intestinal barrier [106,107]. On the other hand, as observed in the interaction between *Neisseria meningitidis* and its filamentous phage MDAφ, the presence of phages may increase bacterial binding to gut epithelial cells and enhance host–cell colonization [108].

Moreover, phages may impact CNS function by modulating both innate and adaptive immune responses [11,25,109,110]. Phages can modulate innate immunity through phagocytosis and cytokine response. Phages that have entered the bloodstream and other tissues can come into contact with immune system cells and be phagocytized by macrophages, dendritic cells, or neutrophils. Phagocytosis can occur while phages are still bound to their bacterial hosts or directly on phages, either through binding to surface molecules or receptors or by entering via mechanisms similar to phage transcytosis [111,112,113]. Emerging evidence suggests that phages can modulate immune cell function following their uptake. For instance, they may enhance the phagocytosis of bacteria by macrophages [114] and, in some contexts, reduce the production of reactive oxygen species by phagocytic cells [115,116]. Moreover, phagocytosed phages have been shown to induce cytokine production, including both pro-inflammatory (e.g., IL-6, TNF-α, IL-1β) and anti-inflammatory (e.g., IL-10, IL1RN) mediators [39,117,118,119]. Phages can also influence adaptive immunity by modulating T-cell polarization and promoting B-cell antibody production. Indeed, it has been demonstrated that phages can modulate the inflammatory response by inhibiting or stimulating T-cell activity [39,120,121]. Furthermore, the internalization of phages by antigen-presenting cells can trigger B cell activation and subsequently produce phage-specific antibodies and an inflammatory response [122,123,124].

Finally, studies suggest phages may translocate from the intestinal tract to other areas, including the CNS [22,25,88]. In particular, recent research detected viral particles, with phages predominant, in the cerebrospinal fluid of healthy individuals, suggesting that CNS regions may harbor phages [125]. Of note, phages have been shown to cross the epithelial cell barrier (in particular neural endothelial cells) via the transcellular route or through infected bacteria phagocytosed by immune system cells (“Trojan horse” route), suggesting a potential mechanism for their migration outside the gut [126,127,128]. However, it remains unclear whether these phages play a role or if their presence in the CNS has no effect [88].

The interaction between phages and CNS has been linked to both beneficial and harmful effects [88]. For example, a recent study showed that higher levels of Caudovirales phages (the class has been abolished and now replaced with the class Caudoviricetes [129,130]), particularly those from the Siphoviridae family, were associated with better performance in executive functions and verbal memory in humans [131]. Conversely, increased levels of Microviridae were linked to poorer cognitive performance [131]. Moreover, microbiota transplantation from human donors with increased levels of Siphoviridae phages led to memory improvement and upregulation of memory-promoting genes in mice [131].

The role of phages has also been investigated concerning CNS diseases. While on one hand, phage dysbiosis has been associated with pathological conditions, on the other hand, recent laboratory techniques such as phage display technology indicate the use of phages as a potential diagnostic and therapeutic approach [43,88,89,132,133]. Although phage engineering is a potential strategy to combat certain CNS neoplasms, it is still rarely mentioned in the literature as a treatment for nervous system disorders [88].

Below, we provide examples of the involvement of phages in CNS pathologies and their potential use for therapeutic purposes. The studies showing a possible correlation between phages and pathologies of the CNS are outlined in Table 1.

### 5.1. Phages and Schizophrenia

Schizophrenia is a complex, widespread neuropsychiatric disorder with a global prevalence [143]. Recent findings show that schizophrenia and related psychiatric disorders are defined by both genetic factors and environmental conditions [144]. Among genetic factors, some genes related to the immune response have been identified as risk factors [145]. Furthermore, schizophrenia is commonly associated with immune dysfunction, typically evidenced by elevated levels of antibodies to exogenous antigens and systemic inflammatory markers [146,147]. Nevertheless, the underlying pathological events leading to the altered inflammatory state observed in individuals with schizophrenia remain unclear.

Recent studies suggested that microbiota-induced inflammation may play a role in psychiatric disorders, including schizophrenia, by promoting systemic inflammation or modulating brain activity via immune/related mechanisms [148,149].

Despite several studies that have reported gut microbiota alterations in patients and animal models with schizophrenia [150,151,152,153], very little is known about the role of the virome in this disease. Recently, Tao and colleagues, besides confirming alterations in the overall community composition of gut bacteria from patients with schizophrenia, performed a Multiple Co-Inertia Analysis (MCIA) to identify specific gut viruses and peripheral metabolites associated with differential gut bacteria [52].

While a significant trans-kingdom correlation between bacteria, bacterial metabolites, and viruses was observed in control subjects, schizophrenic patients showed a drastic reduction in this correlation, mainly driven by Suoliviridae, Rountreeviridae, Pachyviridae, and Schitoviridae [134]. It is known that the bacteriophages Pachyviridae and Schitoviridae are part of the Caudovirales order [154]. Interestingly, bacteriophages of the Caudovirales order have improved cognitive performance in executive function tests in mice and humans [131]. The findings suggest a potential causal pathway where gut bacteriophages may contribute to schizophrenia by influencing gut bacteria and peripheral metabolites. However, our understanding of how gut bacteriophages influence the bacterial community remains limited, as most gut viruses cannot yet be associated with specific bacterial hosts [155].

Moreover, although the gut microbiota remains the primary focus of microbiome research, increasing attention is being given to other microbiomes, including the oropharyngeal microbiota. A study based on whole genome sequencing demonstrated that the oropharyngeal phageome of patients with schizophrenia differs from that of controls, with a significant increase in *Lactobacillus phage phiadh* observed in schizophrenia patients [156]. *Lactobacillus phage phiadh* preferentially infects *Lactobacillus gasseri*, a commensal bacterium with immunomodulatory properties that harbors the oral and gastrointestinal mucosae [157,158].

Human administration of *Lactobacillus gasseri* has demonstrated multiple beneficial effects on immune system function and gastrointestinal health [159]. While its direct role remains unclear, *Lactobacillus phage phiadh* may influence schizophrenia through immune modulation or bacterial interactions.

### 5.2. Phages and Depression

Depression, also known as major depressive disorder (MDD), is a chronic and recurrent neuropsychiatric disorder characterized by anxiety, decreased thinking, and delayed thinking, affecting more than 280 million people worldwide [160]. Despite advancements in understanding the pathophysiology of MDD, the underlying biological mechanisms remain not fully understood and are poorly characterized.

In the last decade, many studies have demonstrated that dysbiosis of gut microbiota participates, in association with immunity, in the pathogenesis of depression [161]. Despite this, the role of gut virome has been poorly explored in MDD.

A recent study using cross-sectional whole-genome shotgun metagenomics analysis showed that patients with MDD had decreased abundances of *Clostridium_*phage_phi8074-B1 and *Klebsiella*_phage_vB_KpnP_SU552A, as well as increased abundance of *Escherichia*_phage_ECBP5, compared with control patients [135]. These three bacteriophages were assigned to the Caudovirales order and are known to shape their corresponding well-known hosts, including *Clostridium sporogenes*, *Klebsiella pneumoniae*, and *Escherichia coli*. According to this, authors also observed an alteration of *Klebsiella pneumoniae* in depressed patients compared to relative controls. These findings suggest that it is reasonable to explore the roles of these phages and their bacterial hosts in the development of MDD. In addition, the authors found a correlation between some bacteriophages with some metabolites, suggesting that these bacteriophages may indirectly affect metabolites by targeting bacterial species [135]. Given that amino acid metabolic dysregulation is a hallmark of the gut environment in MDD [162,163], future research should employ more detailed longitudinal studies to clarify the interactions among gut phages, bacteria, and their associated metabolites.

### 5.3. Phages and Parkinson’s Disease

Parkinson′s disease (PD) is the second most frequent neurodegenerative disorder, characterized by bradykinesia, rigidity, tremor, and gastrointestinal dysfunction. The key neuropathological features of PD are the neuronal degeneration in the substantia nigra, leading to a deficiency of dopamine in the striatum, along with the presence of toxic intracellular inclusions composed of α-synuclein aggregates [164].

Numerous clinical and preclinical studies have demonstrated alterations in intestinal permeability, inflammation, and microbiota composition, reinforcing the role of the gut–brain axis in PD [165]. PD patients show higher levels of bacteria such as *Verrucomicrobia*, *Mucispirillum*, *Porphyromonas*, *Lactobacillus*, and *Parabacteroides*. Additionally, an increase in “pro-inflammatory” bacteria such as Proteobacteria and Gammaproteobacteria has been observed [166,167], along with a significant reduction in butyrate-producing bacteria like *Faecalibacterium prausnitzii* and *Roseburia*, which are crucial for maintaining gut health and anti-inflammatory responses [168,169]. A recent study identified significant changes in the composition of bacteriophages within the gut microbiota of drug-naive PD patients compared to healthy controls. Notably, there were alterations in the phage-to-bacteria ratio, especially within lactic acid bacteria, which are known for their role in dopamine production and regulation of intestinal permeability. The study observed a marked depletion of *Lactococcus* spp. in PD patients, likely driven by an increase in lytic *lactococcal* phages (such as c2-like and 936-like), which are commonly found in dairy products [136].

A recent study revealed significant alterations in the gut phageome of PD patients compared to healthy controls, with metagenomic analysis showing changes in phage abundance, suggesting a potential association with the disease. Phages from the Microviridae and Tectiviridae families were found to be more abundant in PD patients, while phages from the Gokushovirinae subfamily were more abundant in the control group. Moreover, the study found 1621 CRISPR arrays within viral contigs, though only 20 contained cas genes. These phage-encoded CRISPR systems were short and often targeted phages infecting *Faecalibacterium* and *Escherichia* species, suggesting complex interactions between the phages and the bacteria in the gut. Importantly, a machine learning model based on phage abundance data was able to distinguish PD patients from controls with moderate accuracy, highlighting the promising role of the gut virome, and specifically its phage components, as a novel and non-invasive biomarker for PD diagnosis and progression monitoring [137].

In addition to their potential as biomarkers, bacteriophages have also been investigated for the development of PD targeted treatments. In a recent preclinical study, the use of phage display technology was used to identify highly specific and high-affinity antibodies (P21 and P22) with potent inhibitory effects on α-synuclein aggregation in vitro. Moreover, the systemic administration of P21 and P22 antibodies in a mouse model of PD significantly reduced α-synuclein pathology, prevented nigrostriatal neurodegeneration, and improved motor function [170].

In another study, the therapeutic potential of bacteriophages was explored in targeting *Enterococcus faecalis*, a bacterium that converts orally administered levodopa (L-DOPA) into dopamine via bacterial tyrosine decarboxylase (TDC), in an MPTP mouse model of PD. Bacteriophages PBEF62, PBEF66, and PBEF67 were shown to enhance the therapeutic efficacy of L-DOPA by selectively eliminating *E. faecalis* cells, reducing the copies and transcripts of the TDC gene responsible for converting L-DOPA into dopamine in the gut, before it can act on the brain. This suggests that bacteriophages could serve as a valuable adjunct to L-DOPA therapy for PD [171].

Therefore, bacteriophage therapy represents a promising and targeted approach to modulate the gut microbiota and improve L-DOPA efficacy in PD; however, challenges such as phage specificity, stability in the gastrointestinal environment, and long-term safety must be carefully addressed before clinical application.

### 5.4. Phages and Alzheimer’s Disease

Alzheimer’s disease (AD) is a progressive, fatal, and currently untreatable neurodegenerative disorder characterized by memory and cognitive impairment. It is marked by a slow accumulation of amyloid-beta (Aβ) plaques and tangles of hyperphosphorylated tau neurofibrils in the CNS, contributing to neuroinflammation and neuronal loss [172].

Recent studies show that anti-Aβ antibodies can reduce brain amyloid levels and slow the progression of mild dementia by approximately 30% [173]. However, alternative strategies are needed to improve treatment efficacy. The gut–brain axis has attracted increasing interest in AD, as mounting evidence links gut microbiota alterations to AD progression. While most human and animal studies have focused on bacterial populations [174], some have also reported differences in gut phage communities between AD patients and healthy controls. Interestingly, amyloid-positive AD patients presented lower alpha diversity, indicating reduced bacteriophage richness. The Siphoviridae family and *Lactococcus* phages, including bIL285, *Lactococcus* phage bIL286, *Lactococcus* phage bIL309, *Lactococcus* phage BK5-T, *Lactococcus* phage BM13, *Lactococcus* phage P335, *Lactococcus* phage phiLC3, *Lactococcus* phage r1t, *Lactococcus* phage Tuc2009, *Lactococcus* phage ul36, and *Lactococcus* virus bIL67, were significantly reduced in amyloid-positive AD patients [139]. A recent study found that Caudovirales bacteriophages, particularly those from the Siphoviridae family, are associated with improved executive function and memory in flies, mice, and humans [131]. The study also identified a strong correlation between Caudovirales levels and choline, a key source of 1C units of folate, which was shown to block the production of Aβ plaques [175].

Regarding *Lactococcus* phages, their role and interaction with *Lactococcus* bacterial species warrant further investigation, particularly in AD. For instance, *Lactococcus*, a genus of lactic acid bacteria, is known for its production of lactic acid, and it plays a dual role in the brain of AD patients. On one hand, long-term memory counts on the transfer of lactic acid from astrocytes to neurons; on the other hand, excessive accumulation of lactic acid in the brain can promote the deposition of Aβ proteins and lead to an overproduction of lactic acid within neurons. Excess of lactic acid contributes to lowered pH, mitochondrial dysfunction, neuronal death, and overall brain impairment. Consequently, the bidirectional effect of lactic acid underscores the importance of both its production and regulation in the pathogenesis of AD [176,177,178].

Preclinical studies have also observed changes in gut microbiota composition associated with AD progression. For instance, a recent study examined phage alterations in a preclinical AD model, the APP/PS-1 mice [138]. This longitudinal and multi-kingdom gut microbiome analysis by Zhang and colleagues revealed that APP/PS-1 male mice had higher levels of *Lahndsivirus rarus* at 3 months, as well as more *Lactobacillus* prophage Lj771, *Lactobacillus* phage KC5a, and *Lactobacillus* phage phi jlb1 at 6 months, compared to wt mice of the same age and sex. Conversely, APP/PS-1 male mice showed lower *Enquatrovirus* N4 and *Eneladusvirus* BF levels at 3 and 4 months. These *Lactobacillus* phages may reduce the abundance of *Lactobacillus* species, which have accumulated significant attention for their potential neuroprotective effects in AD. In particular, evidence suggests that supplementation with various *Lactobacillus* strains can reduce neuroinflammation and oxidative stress, lowering pro-inflammatory cytokines and enhancing antioxidant mechanisms [179,180]. These bacteria may reduce Aβ deposition by modulating the expression of amyloid precursor proteins [181], positively influencing gut microbiota composition, improving intestinal barrier integrity, and producing short-chain fatty acids characterized by anti-inflammatory and neuroprotective properties [182]. Moreover, a randomized, double-blind trial evaluated the effects of probiotic supplementation, *Lactobacillus* and *Bifidobacterium*, on cognitive function, oxidative stress, and inflammation parameters in patients with AD. This study demonstrated that probiotic-treated individuals exhibited significant improvements in cognitive performance, specifically the mini-mental state examination, with reductions in oxidative stress and inflammation markers compared to placebo controls. These findings reinforce the connection between gut microbiota composition and AD pathology, suggesting that targeted modulation of the gut microbiota could be a promising strategy to slow cognitive decline in AD patients [183]. M13 bacteriophages have shown potential in treating neurodegenerative diseases like AD and PD due to their filamentous structure, which allows them to bind selectively to protein aggregates such as beta-amyloid plaques and alpha-synuclein. In vitro studies have demonstrated their ability to disaggregate amyloid plaques, reducing their accumulation in the brain [184]. Phage display technology further expands therapeutic possibilities by enabling the development of peptide inhibitors targeting Aβ and tau aggregation and biocompatible metal-chelating agents [185].

Additionally, this technology has facilitated the identification of peptides capable of crossing the BBB, improving CNS drug delivery. For instance, cyclic SLS and TGN peptides have successfully delivered phages, drugs, and nanoparticles to the brain and amyloid plaques in AD models [185]. These findings and increasing interest in microbiota modulation via bacterial and phage-based approaches highlight promising new avenues for AD diagnosis and treatment, though further studies are needed.

### 5.5. Phages and Huntington’s Disease

Huntington’s disease (HD) is an inherited neurodegenerative disorder characterized by a combination of symptoms including motor dysfunction (most commonly chorea), cognitive impairments (such as deficits in attention and emotion recognition), and neuropsychiatric symptoms (including apathy) [186]. An expanded CAG trinucleotide causes HD repeat within the *HTT* gene, which encodes the huntingtin protein [186]. The mutations produce huntingtin proteins with long polyglutamine tracts, leading to neuronal dysfunction and death [186]. Although treatments are available to manage symptoms and improve quality of life, no treatments can alter the HD progression [187].

Recent studies have indicated gut microbiota dysbiosis in both HD preclinical models and patients [188,189,190,191] and suggested that gut microbiota modulation may represent a potential intervention for this disease [192,193,194]. However, to our knowledge, the role of phages in the microbiota dysbiosis has not been investigated.

Phage display technology has been employed to screen for peptides that bind to polyglutamine sequences to prevent HD onset or slow HD progression [195,196,197]. Nonetheless, the bacteriophage component of the gut microbiota in HD patients remains largely uncharacterized. Addressing this gap through future studies could provide novel insights into the relation between the gut microbiome and HD, and potentially open new avenues for phage-based therapeutic strategies.

### 5.6. Phages and Amyotrophic Lateral Sclerosis

Amyotrophic lateral sclerosis (ALS) is a progressive and currently incurable disease characterized by the degeneration of motor neurons, leading to muscle weakness and wasting. Approximately 50% of patients also present extra-motor symptoms, including behavioral and language alterations [198]. The disease is marked by significant pathological heterogeneity, which results in variability in clinical symptoms. ALS can be classified into two types: familial ALS, caused by inherited genetic mutations (e.g., SOD1), and sporadic ALS, developed without a clear genetic cause, and representing the most common form of the disease [199].

Unfortunately, none of the currently available treatments have proven capable of effectively curing the disease; rather, they focus primarily on alleviating symptoms [200]. Interventions targeting the gut microbiota, such as antibiotic treatments, probiotic supplementation, and phage therapy, have shown promise in reducing inflammation, slowing neuronal degeneration, and potentially delaying disease progression. In particular, recent studies have shown that the use of *A. muciniphila* supplementation alleviated ALS symptoms, whereas *Ruminococcus torque* and *P. distasonis* exacerbated these symptoms in Tg mice [201]. Moreover, phage-targeted strategies have shown promise in models of ALS. For instance, using a phage display platform, single-chain variable fragment antibodies raised against SOD1 significantly improved disease outcomes in G93A mutant SOD1 mice. These antibodies prolonged disease duration and survival, reduced motor neuron loss, and decreased SOD1 aggregation [202]. Numerous studies have explored bacterial alterations in ALS patients [203], but variations in the gut phageome between ALS patients and healthy individuals remain largely unexplored. Nonetheless, recent evidence suggests that orally administered phage therapies can modulate the gut microbiota without disrupting microbial symbiosis, reduce intestinal inflammation, and prevent antibiotic resistance in a mouse model colonized by *K. pneumoniae* associated with inflammatory bowel disease. Furthermore, phage therapy against *K. pneumoniae* was conducted using a human artificial gut model and healthy volunteers, demonstrating gastric acid-dependent phage resilience, safety, and viability in the lower intestinal tract [204]. These findings support the rationale for developing phage-based therapeutic strategies for ALS, including familial ALS, potentially in combination with SOD1-targeted antibodies [81,205].

### 5.7. Phages and Multiple Sclerosis

Multiple sclerosis (MS) is a chronic inflammatory disease of the CNS characterized by demyelination that leads to neuronal dysfunction and eventually cell death [206]. The clinical features of MS are varied and depend on the areas of the CNS affected by the pathology. These symptoms can include sensory and motor problems, visual disturbances, cognitive impairment, and emotional changes [207]. Although therapeutic advances can slow the disease progression, MS is non-curable to date [208].

Different preclinical and clinical investigations found associations between MS status and gut microbiota composition [209,210,211,212,213]. A recent study analyzed the gut virome of healthy subjects and patients affected by autoimmune diseases, including MS, rheumatoid arthritis, and systemic lupus erythematosus (SLE) [140]. Unlike rheumatoid arthritis and SLE, the authors did not observe any significant differences in virome composition between controls and subjects affected by MS, which, according to the authors themselves, may be due to the small sample size of MS patients enrolled in the study [140].

Phage display technology has been extensively utilized to produce peptides targeting inflammatory sites in the CNS for diagnostic or therapeutic purposes in MS [214,215,216,217,218]. However, no phage-based therapies have yet been tested or validated for the treatment of this disease. In this context, future studies involving larger cohorts of MS patients would be valuable to better characterize the gut virome and investigate the potential role of specific phages as therapeutic agents.

### 5.8. Phages and Stroke

Stroke is a sudden cerebrovascular event causing brain tissue damage, mainly triggered by cerebral ischemia or hemorrhage due to blocked or ruptured blood vessels. This disease has high rates of mortality and disability [15,219,220].

Interestingly, stroke has been associated with alterations in the gut virome [15]. In the mouse middle cerebral artery occlusion (MCAO) stroke model, significant changes in fecal virome taxa and at the strain level were observed. Alpha diversity was decreased in the stroke model, as was the abundance of Siphoviridae_u_t and *Lactobacillus prophage* Lj771_u_t compared to the control group [141]. Moreover, the composition of the virome varied substantially, with the relative abundance of Bacteroides phage B40_8 and Cronobacter phage CS01 significantly elevated in stroke patients compared to healthy individuals [15,142]. Despite various treatments, including surgery, pharmacological, and interventional, these approaches still have limitations and side effects [219]. Researchers have explored new treatment modalities for post-stroke recovery. The use of bacteriophages to promote cellular adhesion, differentiation and proliferation, neuron survival, and axonal regeneration, has been attracting increasing attention in the last decades [219].

Hong and colleagues utilized a phage peptide library to screen homing peptides to ischemic stroke tissue in a rat MCAO stroke model. The CLEVSRKNC-phage specifically targeted ischemic stroke tissue, indicating that this peptide could be valuable when conjugated with neuroprotective agents or nanoparticles carrying therapeutic drugs. Additionally, it may aid in monitoring drug efficacy while detecting apoptotic neuronal cells [221].

Liu and colleagues investigated the potential of the M13 phage in regenerating brain tissue within the stroke cavity. The injection of neural stem cell-seeded R-phage-MPs into ischemic regions of rat brains triggered a cascade of reparative events, promoting brain tissue recovery post-stroke. R-phage was prepared at concentrations of 1 × 10^5^, 1 × 10^9^, and 1 × 10^13^ PFU/mL to generate low-, medium-, and high-R-phage-MPs, respectively. Both medium- and high-R-phage-MPs demonstrated significantly greater brain tissue regeneration and functional improvement compared to low-R-phage-MPs. They observed the proliferation and migration of immature neuroblasts in the subventricular zone, along with a decrease in scar tissue thickness due to reduced inflammation and limited astrocyte migration. The regenerated neurons were surrounded by newly formed brain tissue, rich in mature and well-developed nerve fibers [222].

Another approach involves using bacteriophages to treat antibiotic-resistant bacterial infections. *Enterococcus faecalis*, a pathogen capable of causing pneumonia and bacteremia in stroke patients, can be effectively targeted with a combination of antibiotics and the *E. faecalis* bacteriophage Vb_EfaM_LG1. This strategy not only disrupts biofilms efficiently but also helps prevent the development of phage resistance [223].

### 5.9. Phages and Epilepsy

According to the International League Against Epilepsy, epilepsy is a neurological disorder characterized by recurrent, unprovoked seizures resulting from abnormal electrical activity in the brain [224]. Various factors can disrupt the excitatory/inhibitory balance in neurons [225,226], including alterations in the gut microbiome, which has been hypothesized to play a significant role in epilepsy and seizures [226,227].

In the literature, it is possible to find correlations between bacterial or viral infections and epilepsy or epileptic seizures, but the role of bacteriophages remains unclear. Roch and colleagues conducted a metagenomic study to identify viral DNA and RNA sequences in patients with onchocerciasis-associated epilepsy (OAE). Various bacteriophages were detected in all cases and controls; however, none of the identified viral sequences were enriched in OAE cases [228].

Brain microbiome diversity and abundance were investigated in pathologically normal and abnormal brains from individuals with HIV/AIDS (HIV), other disease controls (ODC), and cerebral surgical resections for epilepsy (SURG). The majority of bacterial sequences identified in all patient samples belonged to the Proteobacteria phylum, with the highest similarity to the α-proteobacteria class (70%). Furthermore, most bacteriophage sequences identified matched Proteobacteria-tropic phage sequences; however, bacteriophage sequences were not detected in the SURG samples [229].

It is known that neurocysticercosis, an infection of the central nervous system caused by *Taenia solium*, results in a diverse range of neurological complications, including epilepsy in endemic areas. Phage display was used as a tool to identify peptides that mimic an antigen of *T. solium*. The most promising peptide was synthesized without the phage and tested as a potential diagnostic biomarker for neurocysticercosis [230].

## 6. Conclusions

The incomplete list of studies cited in this review indicates, on one hand, the close connection between phages and CNS health, and on the other hand, underscores the limited exploration this topic has received so far. For these reasons, we encourage future research to investigate the correlation between phage dysbiosis and CNS disorders and identify novel phage-based diagnostic and therapeutic interventions for such conditions.

## Figures and Tables

**Table 1:** The table outlines the studies investigating a correlation between phage composition and CNS pathologies. No studies have been identified addressing the relation between phages and ALS, HD, or epilepsy.

Pathology	Model	Findings	Reference
Schizophrenia	human (patients)	↓ Suoliviridae, Rountreeviridae Pachyviridae, and Schitoviridae-bacterial correlation profile	[134]
Depression	human (patients)	↓ *Clostridium_*phage_phi8074-B1 *↓ Klebsiella_*phage_vB_KpnP_SU552A *↑ Escherichia_*phage_ECBP5	[135]
PD	human (patients)	↓ *Lactococcus* spp. ↑ Microviridae ↑ Tectiviridae	[136,137]
AD	mice (APP/PS-a model)	↑ *Lahndsivirus rarus* (at 3 months) ↑ *Lactobacillus* prophage Lj771 (at 6 months) ↑ *Lactobacillus* phage KC5a (at 6 months) ↑ *Lactobacillus* phage phi jlb1 (at 6 months) ↓ *Enquatrovirus* N4 (at 3 and 4 months) ↓ *Eneladusvirus* BF (at 3 and 4 months) ↓ *Lactococcus*	[138]
	human (patients)	↓ Siphoviridae ↓ *Lactococcus*	[139]
MS	human (patients)	no difference between healthy subjects and patients	[140]
Stroke	mice (MCAO model)	↓ Siphoviridae_u_t ↓ *Lactobacillus* prophage Lj771_u_t	[141]
	human (patients)	↑ *Bacteroides* phage B40_8 ↑ *Cronobacter* phage CS01	[15,142]

## Data Availability

Not applicable.

## Abbreviations

The following abbreviations are used in this manuscript:

AD	Alzheimer’s disease
ALS	Amyotrophic lateral sclerosis
ANS	Autonomic nervous system
Aβ	Amyloid-beta
BBB	Blood–brain barrier
CNS	Central nervous system
ENS	Enteric nervous system
MGBA	Microbiota–Gut–brain axis
HD	Huntington’s disease
HPA	Hypothalamic–pituitary–adrenal
i.v.	Intravenous
L-DOPA	Orally administered levodopa
MCAO	Middle cerebral artery occlusion
MDD	Major depressive disorder
MS	Multiple sclerosis
OAE	Onchocerciasis-associated epilepsy
PD	Parkinson disease
ROS	Reactive oxygen species
SCFAs	Short-chain fatty acids
SLE	Systemic lupus erythematosus
SURG	Surgical resections for epilepsy
TDC	Tyrosine decarboxylase
